# Progress and Prospects on Vaccine Development against SARS-CoV-2

**DOI:** 10.3390/vaccines8020153

**Published:** 2020-03-29

**Authors:** Jinyong Zhang, Hao Zeng, Jiang Gu, Haibo Li, Lixin Zheng, Quanming Zou

**Affiliations:** 1 National Engineering Research Center of Immunological Products, Department of Microbiology and Biochemical Pharmacy, College of Pharmacy and Laboratory Medicine, Third Military Medical University, Chongqing 400038, China; zhangjy198217@126.com (J.Z.); zeng1109@163.com (H.Z.); jianggu2012@163.com (J.G.); lihaibo@tmmu.edu.cn (H.L.); 2 Laboratory of the Immune System Biology, National Institute of Allergy and Infectious Diseases, National Institutes of Health, Bethesda, MD 20892, USA

**Keywords:** SARS-CoV-2, vaccine, adjuvant, animal model

## Abstract

In December 2019, the outbreak of pneumonia caused by a novel coronavirus, severe acute respiratory syndrome coronavirus 2 (SARS-CoV-2), has led to a serious pandemic in China and other countries worldwide. So far, more than 460,000 confirmed cases were diagnosed in nearly 190 countries, causing globally over 20,000 deaths. Currently, the epidemic is still spreading and there is no effective means to prevent the infection. Vaccines are proved to be the most effective and economical means to prevent and control infectious diseases. Several countries, companies, and institutions announced their programs and progress on vaccine development against the virus. While most of the vaccines are under design and preparation, there are some that have entered efficacy evaluation in animals and initial clinical trials. This review mainly focused on the progress and our prospects on field of vaccine development against SARS-CoV-2.

## 1. Introduction

In December 2019, a cluster of patients with pneumonia surfaced in Wuhan, China. The culprit was quickly identified as a beta-coronavirus that has never been reported before, and the virus was named by WHO as SARS-CoV-2 [1,2]. By March 26, 2020, more than 80,000 people in China had been confirmed to be infected with the pathogen, and among them, 3293 people died from the infection. Under the aggressive control measures implemented by the Chinese government, the severe domestic epidemic seems to have been effectively contained. However, a global outbreak has caused more than 460,000 confirmed cases in more than 190 other countries, making the SARS-CoV-2 pandemic a general public health event that has stirred up worldwide attention.

**Figure 1:**
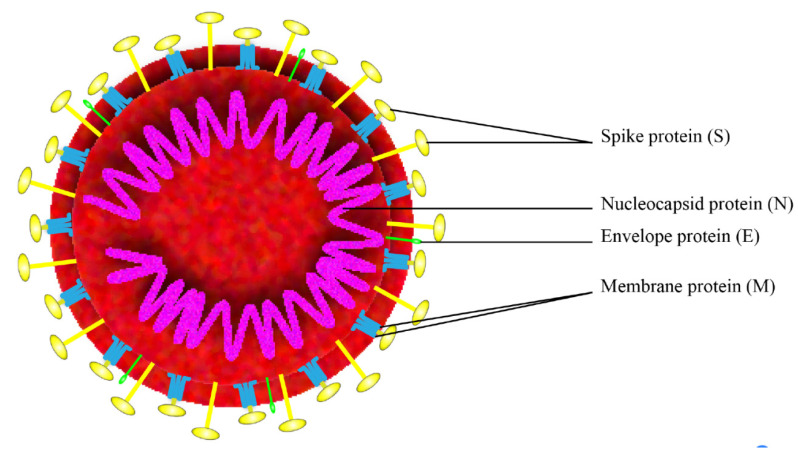
Schematic of the overall structure of SARS-CoV-2.

SARS-CoV-2 is a positive-strand RNA virus that belongs to the group of Betacoronaviruses. The genome of SARS-CoV-2 is approximately 29,700 nucleotides long and shares 79.5% sequence identity with SARS-CoV [3], it has a long ORF1ab polyprotein at the 5′ end, which encode 15 or 16 non-structural proteins. The 3′ end of the genome encodes 4 major structural proteins, including the spike (S) protein, nucleocapsid (N) protein, membrane (M) protein, and the envelope (E) protein [4], as shown in Figure 1. SARS-CoV-2 binds to the receptor angiotensin converting enzyme 2 (ACE2) on host cell for the virus entry and subsequent pathogenesis [5], resulting in severe respiratory illness with symptoms of fever, cough, and shortness of breath, and severe cases can be fatal [6].

Vaccines are the most effective and economical means to prevent and control infectious diseases [7]. The development of an effective vaccine against SARS-CoV-2 infection is urgently required. So far, more than 40 pharmaceutical companies and academic institutions worldwide have launched their programs on vaccine development against SARS-CoV-2. Herein, we will summarize the recent progress on this vaccine development worldwide, in particular, on candidate antigens, adjuvants, evaluation models, and technologies used in related research.

## 2. Antigen Selection

### 2.1. Whole Cell Antigens

The whole cell antigens (WCA) contain all the elements of the virus, including proteins, lipids, polysaccharide, nucleic acids, and some other components. WCA has been used for developing whole-cell killed and live-attenuated vaccines [8,9]. Given the complex compositions of WCA, it is unavoidable to face more difficulties in quality control and consistency evaluation. So far, several institutions have successfully isolated the virus strains of SARS-CoV-2 and started whole-cell killed or live-attenuated vaccine development. However, research on the type of vaccine requires stringent screening for obtaining strains with assured low or no pathogenicity [10].

### 2.2. Spike Protein (S Protein)

S protein is currently the most promising antigen formulation for SARS-CoV-2 vaccine research. First, it is surface exposure and thus is able to be directly recognized by host immune system [11]. Second, it mediates the interaction with host cell by binding to the receptor ACE2, which is essential for subsequent virus entry to target cells and causing subsequent pathogenicity [11,12]. Finally, the homologue proteins were already used for vaccine development against SARS-CoV and MERS-CoV, and were proved to be effective [13,14,15,16,17].

The monomer of S protein from SARS-CoV-2 contains 1273 amino acids, with a molecular weight of about 140 kDa. Self-association naturally assembles the S protein into a homo-trimer, typically similar to the first class of membrane fusion protein (Class I viral fusion protein). The S protein contains two subunits (S1 and S2). The S1 subunit can be further defined with two domains termed the N-terminal domain (NTD) and the C-terminal domain (CTD). The receptor binding domain (RBD) is located in the CTD. S2 subunit contains the basic elements required for membrane fusion, including an internal membrane fusion peptide (FP), two 7-peptide repeats (HR), a membrane proximal external region (MPER), and a trans-membrane domain (TM) [18]. Recently, the structure of the SARS-CoV-2 S trimer in the pre-fusion conformation and the RBD domain in complex with ACE2 has been successfully determined [11,12], which has provided valuable information for vaccine design based on this protein. So far, the potential fragments of S protein for use as antigens in vaccine development include the full-length S protein, the RBD domain, the S1 subunit, NTD, and FP.

#### 2.2.1. The Full-Length S Protein

Full-length proteins are likely to maintain the correct conformation of the protein, capable of providing more epitopes and exhibiting higher immunogenicity. Pallesen et al. [19] demonstrated that higher titer of neutralizing antibodies in BALB/c mice immunized with recombinant prefusion MERS-CoV S protein. Another research confirmed that S protein produced in baculovirus insect cells was able to assemble into nanoparticles. Mice immunized with these nanoparticles formulated with alum adjuvant that produced high titer of neutralizing antibodies [20]. Muthumani et al. [21] reported that DNA vaccine encoding MERS-CoV S protein was immunogenic in mice, camels, and rhesus macaques. Animals immunized with the DNA vaccine show reduced typical clinical symptoms including pneumonia during the infection. So far, Clover Biopharmaceuticals had announced that they have constructed a SARS-CoV-2 S protein trimer vaccine (S-Trimer) by using its patented Trimer-Tag© technology, and this vaccine will be produced via a rapid mammalian cell-culture based expression system, further preclinical safety and analysis will be launched within the next 6–8 weeks.

#### 2.2.2. RBD

Since the RBD of S protein directly interacts with the ACE2 receptor on host cells, RBD immunization induced specific antibodies may block this recognition and thus effectively prevent the invasion of the virus. As a matter of fact, most of SARS-CoV-2 subunit vaccines currently under development use RBD as the antigen. Moreover, the RBD domain was also used in the development of SARS-CoV and MERS-CoV vaccines. For example, studies have demonstrated that recombinant RBD consists of multiple conformational neutralizing epitopes that can induce high titer of neutralizing antibodies against SARS-CoV [22]. Lan et al. [23] reported that Rhesus macaques immunized with the recombinant RBD formulated with alum adjuvant could produce neutralizing antibodies, in association with observed mitigation of the clinical symptoms during MERS-CoV infection. Nyon et al. [24] also reported that hCD26/DPP4 transgenic mice immunized with RBD fused to Fc elicited neutralizing antibodies and were capable of protecting MERS-CoV infection. Further, RBD domain is relatively conserved as compared with S1 subunit and was reported to contain multiple conformational neutralizing epitopes [25], making it more suitable for vaccine development.

#### 2.2.3. NTD

Similar to RBD, the N-terminal domains (NTD) of S protein from several coronaviruses were reported to show carbohydrate receptor-binding activity. For example, the NTD of spike protein form transmissible gastroenteritis virus (TGEV) was reported to bind sialic acid via NTD [26]. The carbohydrate-binding properties of IBV M41 strain are also related to the NTD of the S protein [27]. Thus, this domain is also a candidate antigen for vaccine development. One study reported that rNTD of S protein from MERS-CoV induced potent cellular immunity and antigen-specific neutralizing antibodies in mice and was protective against the viral challenge [28]. There is a report that a mAb that binds to the N-terminal domain (NTD) of the MERS-CoV S1 subunit showed efficient neutralizing activity against the wild-type MERS-CoV strain EMC/2012 [29], this result showed that NTD specific antibodies are functional in neutralization. However, as the genomes of coronaviruses are highly variable, it is better to use antibodies targeting different epitopes to avoid immune escape of the virus. Although the function of S1-NTD of SARS-CoV-2 has not been elucidated, it may also be involved in the binding of certain receptors and can also serve as a candidate antigen.

#### 2.2.4. S1 Subunit

The S1 subunit, which contains both RBD and NTD described above, is mainly involved in the S protein binding to the host receptor. It is also widely used in vaccine development. Wang et al. [30] reported that MERS-CoV S1 protein formulated with MF59 adjuvant protected hDPP4 transgenic mice against lethal virus challenge, and the protection correlated well with the neutralizing antibody titer. Adney et al. [31] confirmed that immunization with adjuvanted S1 protein reduced and delayed virus shedding in the upper respiratory tract of dromedary camels and complete protection was observed in alpaca against MERS-CoV challenge.

#### 2.2.5. FP

The FP domain of the S2 subunit is involved in the membrane fusion of the virus, which is also a key step in viral pathogenicity [32]. Therefore, it may also serve as a vaccine candidate antigen. At present, Tianjin University has constructed an RBD-FP fusion protein, and high titer of antibodies was detected in mice immunized with this fusion protein, and the efficacy is under evaluation.

### 2.3. Nucleocapsid Protein (N Protein)

The N protein is the most abundant protein in coronavirus, and it is normally highly conserved, with a molecular weight of about 50 kDa. N protein has multiple functions including formation of nucleocapsids, signal transduction virus budding, RNA replication, and mRNA transcription [33]. This protein was reported to be highly antigenic, 89% of patients who developed SARS, produced antibodies to this antigen [34]. DNA vaccine encoding SARS-CoV N protein generated strong N-specific humoral and cellular immune responses in vaccinated C57BL/6 mice, and was capable of significantly reducing the titer of challenging vaccinia virus [35]. Meanwhile, some other researchers reported that the N protein of avian infectious bronchitis virus is associated with the induction of CTLs that correlated with a decrease in clinical signs and viral clearance from lungs, suggesting cellular response is essential in N protein mediated protection [36,37]. In contrast, another research indicated that the N protein immunization did not make a significant contribution to a neutralizing antibody response and provided no protection to infection in hamsters [38]. These results suggest that there is controversy about whether this protein could be used for vaccine development. Anyhow, there is no doubt that it can be used as a marker in diagnostic assays due to its high immunogenicity.

### 2.4. Membrane Protein (M Protein)

M protein is a trans-membrane glycoprotein with a molecular weight of about 25 kDa and is involved in virus assembly, and this protein is the most abundant protein on the surface of SARS-CoV [39]. It was reported that immunization with the full length of M protein is able to elicit efficient neutralizing antibodies in SARS patients [40]. Immunogenic and structural analysis also indicated that the trans-membrane domain of the M protein contains a T cell epitope cluster that is able to induce a strong cellular immune response [41]. M protein is also highly conserved in evolution among different species [39], therefore it may be used as a candidate antigen for developing SARS-CoV-2 vaccine.

### 2.5. Envelope Protein (E Protein)

Compared with S, N, and M protein, E protein is not suitable for use as an immunogen. For one reason, it consists of 76–109 amino acids in different coronaviruses with channel activity, thus the immunogenicity is limited. Studies have shown that SARS-CoV E protein is an important virulence factor, and the secretion of inflammatory factors IL-1β, TNF, and IL-6 are significantly reduced after knocking out E protein [42].

## 3. Different Types of SARS-CoV-2 Vaccines under Development

### 3.1. Whole-Cell Killed and Live-Attenuated Vaccines

Whole-cell killed or live-attenuated vaccines present multiple antigenic components to the host and can thus potentially induce diverse immunologic effectors against pathogen [43]. They are traditional vaccines with mature preparatory technology, and may become the first SARS-CoV-2 vaccine put into clinical applications. Currently, several research institutions have started related research. The Chinese Centers for Disease Control and Prevention, Wuhan Institute of Virology, Chinese Academy of Sciences, Zhejiang University, and several other institutions have successfully isolated the virus strains of SARS-CoV-2 and started relevant vaccine development. Besides, Codagenix, Inc. announced the collaboration with the Serum Institute of India, Ltd. to develop a live-attenuated vaccine against SARS-CoV-2. They use viral deoptimization to synthesize “rationally designed” live-attenuated vaccines. This technology starting with the sequence of the viral genome and allows for the rapid generation of multiple vaccine candidates against the virus.

### 3.2. Subunit Vaccines

Subunit vaccines include one or more antigens with strong immunogenicity capable of efficiently stimulating the host immune system. In general, this type of vaccine is safer and easier to produce, but often requires the addition of adjuvants to elicit a strong protective immune response. So far, several institutions have initiated programs on the SARS-CoV-2 subunit vaccine, and almost all of them use the S protein as antigens. For example, the University of Queensland is developing a subunit vaccine based on the “molecular clamp” technology [44]. Clover Biopharmaceuticals Inc. revealed that they are developing a vaccine candidate against SARS-CoV-2 using the “Trimer-Tag” technology [45], and the trimeric S protein subunit vaccine candidate was produced via a mammalian cell expression system. Novavax, Inc. announced that they had produced multiple nanoparticle vaccine candidates based on S protein, and now is assessing efficacy in animal models to identify an optimal vaccine candidate for human testing. Besides, Johnson & Johnson, Pasteur Institute, and Chongqing Zhifei Biological Products Co., Ltd. also started subunit vaccine development against SARS-CoV-2.

### 3.3. mRNA Vaccines

With the development and maturing of mRNA synthesis, modification, and delivery technology, the research on mRNA vaccine has regained attention during the last two decades. mRNA vaccines represent a promising alternative to conventional vaccine approaches because of their high potency, short production cycles, low-cost manufacturing, and safe administration [46]. The procedures of mRNA vaccine development include the selection of antigens, the optimization of sequences, the screening of modified nucleotides, the optimization of delivery systems, the evaluation of immune response and safety test [47]. Particularly, no mRNA vaccine has yet entered the market, thus it may take more time in quality standards establishment and safety evaluation. So far, a SARS-CoV-2 mRNA vaccine (mRNA-1273, encoding S protein) developed by Moderna, has been launched in animal experiments and clinical batch production. It is expected that clinical trials will be conducted on 20–25 healthy volunteers by the end of April. Fudan University is in collaboration with Shanghai Jiaotong University and Bluebird Biopharmaceutical Company to develop a SARS-CoV-2 mRNA vaccine using two different strategies. The first is to use mRNA to express the SARS-CoV-2 S protein and RBD domain, the efficacy of this vaccine is now under evaluation in mice. The second is the use of mRNA to express virus-like particles in vivo. In addition, German biopharmaceutical company CureVac AG, Stermirna Therapeutics, BDGENE Therapeutics, Guanhao Biotech, ZY Therapeutics Inc., CanSino Biologics Inc., Baylor College of Medicine, University of Texas, Tongji university also announced their progress on mRNA vaccine development against SARS-CoV-2.

### 3.4. DNA Vaccine

DNA vaccines are typically comprised of plasmid DNA molecules that encode one or more antigens. They are superior to mRNA vaccines in the formulations needed for stability and delivery efficiency, nevertheless they need to enter the nucleus that may bring in the risk of vctor integration and mutations in the host genome [48]. So far, two SARS-CoV-2 DNA vaccines are under development. Inovio Pharmaceuticals developed a DNA vaccine candidate termed INO-4800, which is in preclinical studies and will soon enter phase I clinical trials. Applied DNA Sciences Subsidiary, LineaRx, and Takis Biotech collaborated for the development of a linear DNA vaccine candidate against SARS-CoV-2, which is now in preclinical studies.

### 3.5. Live Vector Vaccines

Live vector vaccines are live viruses (the vector) that express a heterologous antigen(s). They are characterized by combining the strong immunogenicity of live attenuated vaccines and the safety of subunit vaccines, and were widely used to induce cellular immunity in vivo. Related SARS-CoV-2 vaccine research has been carried out by the following institutions. Houston-based Greffex Inc. has completed the construction of SARS-CoV-2 adenovirus vector vaccine with Greffex Vector Platform and should have now moved to animal testing. Tonix Pharmaceuticals announced research to develop a potential SARS-CoV-2 vaccine based on Horsepox Virus (TNX-1800). Johnson & Johnson has adopted the AdVac^®^ adenoviral vector platform [49] for vaccine development.

### 3.6. Synthetic Peptide or Epitope Vaccine

These vaccines contain only certain fragments of intact antigens and are usually prepared by chemical synthesis techniques. They are easier in preparation and quality control. However, the low molecular weight and structural complexity of these vaccines usually result in low immunogenicity, thus structural modifications, delivery systems, and adjuvants are additionally required in the formulation [50]. Currently, researchers from Hong Kong University of Science and Technology screened a set of B and T cell epitopes from S and N proteins of SARS-CoV, these epitopes are highly conserved in SARS-CoV-2 and may help guide experimental efforts towards the development of SARS-CoV-2 vaccines [51]. Generex Biotechnology announced that they are working with third-party groups to generate peptide vaccines against pandemic viruses using the patented NuGenerex Immuno-Oncology Ii-Key technology that uses synthetic peptides in mimic essential protein regions from a virus that is chemically linked to the 4-amino acid Ii-Key to ensure robust immune system activation.

## 4. Means of Efficacy Evaluation

Animal models are essential for preclinical evaluation of the efficacy of vaccines. SARS-CoV-2 is a new pathogen and few animal models are currently available. Recently, one study used the hACE2 transgenic mice infected with SARS-CoV-2 to study the pathogenicity of the virus. hACE2 transgenic mice infected with SARS-CoV-2 resulted in weight loss and virus replication in lung tissue. The typical histopathology of interstitial pneumonia was observed and viral antigens were detected in the bronchial epithelial cells, alveolar macrophages, and alveolar epithelia. This mouse model may facilitate the development of therapeutic drugs and vaccines against SARS-CoV-2 [52]. In addition, the Institute of Zoology of the Chinese Academy of Sciences reported that they observed similar symptoms with human infections in primate macaques, including changes in viral load and computed tomography (CT) images of the lungs. The model has been validated and is about to be applied in drug screening and functional evaluation.

The highly contagious and pathogenicity brings great difficulties for SARS-CoV-2-related research. Virus-like particles (VLPs) are multi-protein structures that mimic the organization and conformation of authentic native viruses but lack the viral genome [53]. They are safe and effective tolls that are widely used in studying the pathogenic mechanisms of virus infection, efficacy evaluation of drugs and vaccines, and drug delivery [54]. For example, the corresponding VLPs have been already used to determine the neutralizing antibodies induced by SARS-CoV and H5N1 vaccines [55,56]. In the absence of animal models, VLPs provide an alternative way to evaluate the efficacy of SARS-CoV-2 vaccines.

## 5. Adjuvant

In addition to live attenuated vaccines and live vector vaccines, adjuvants are required to enhance the immune response in the development of other types of vaccines. In order to accelerate the development of a SARS-CoV-2 vaccine, the preferred adjuvant should be those have been widely used in other marketable vaccines, including (1) classic aluminum adjuvant, aluminum adjuvants enhances the immune response by facilitating phagocytosis and slowing the diffusion of antigens from the injection site. It can efficiently stimulate Th2 immune response upon injection [57]. (2) MF59, MF59 is an oil-in-water emulsion composed of Tween 80, sorbitol trioleate, and squalene, and it has already been used in flu vaccines in Europe and the United States. The mechanism of MF59 is to create a transient immune environment at the injection site, then to recruit immune cells to induce antigen-specific immune responses [58]. (3) Adjuvant system (AS) series adjuvants, which are a series of adjuvants developed by GlaxoSmithKline (GSK), including AS01, AS02, AS03, and AS04. Among them, AS01 is a liposome adjuvant containing 3-O-desacyl-4’-monophosphoryl lipid A (MPL) and saponin QS-21 [59], which has been used in malaria vaccines [60]. AS02 is an oil-in-water emulsifier containing MPL and QS-21 [61]. AS03 is an oil-in-water emulsifier containing alpha-tocopherol, squalene, and Tween 80. It has been used in influenza vaccines [62]. AS04 is an aluminum adjuvant containing MPL and has been used in a human papilloma virus vaccine and hepatitis B virus vaccine [63]. Currently, GSK announced that they would make its established pandemic vaccine adjuvant platform technology available to enhance the development of an effective vaccine against SARS-CoV-2, and agreements have been reached with Clover Biopharmaceutical Inc. and the University of Queensland, Australia.

Because adjuvants were able to regulate the type of immune response, the optimal adjuvant should be selected according to the design of the vaccine. In order to induce a more efficient immune response, a combination of different types of adjuvants could be applied to improve the immune efficacy.

## 6. Safety Concerns

Safety is the most important issue that should be taken into consideration during drug and vaccine development, and some scientists urge that we should not rush to deploy COVID-19 vaccines and drugs without sufficient safety guarantees [64]. For example, although S protein is a promising candidate antigen for vaccine development, it also exhibits other biological activity besides receptor binding and membrane fusion. Previous studies have demonstrated that full-length S protein can cause severe liver damage and may result in enhanced infection [14], which was defined as antibody-dependent enhancement (ADE) [65], and this effect may be probably caused by S protein specific antibodies [66,67]. Further, there is report that the enhanced virulence mediated by the murine coronavirus, mouse hepatitis virus strain JHM, is associated with a glycine at residue 310 of the spike glycoprotein, and this mutation may contribute to the spreading within the central nervous system (CNS) [68]. However, it is not clear which domains and key amino acids in S protein of SARS-CoV-2 are involved in liver damage. Therefore, the full-length S protein may face safety issues when used as an antigen. To solve this problem, more basic research should be carried out on the structure and function of this protein, and mutants of key residues may be introduced in antigen design. Recently, a phase I clinical trial was launched to assess the safety and immunogenicity of mRNA-1273 developed by Moderna (ClinicalTrials.gov Identifier: NCT04283461). However, this type of vaccines has not been approved for use in human beings before, thus the safety is not guaranteed and more assessments should be carried out during clinical trials.

## 7. Prospects

We know very little about SARS-CoV-2. There are more questions than answers for the newly identified virus, including the etiology, epidemiology, structural basis, mechanism of pathogenesis, pathological immune response, etc. Particularly, the cellular and humoral immune response of the host in response to the virus infection that are essential for vaccine design, remains unclear. All these aspects need to be addressed by basic research in the near future for successful vaccine development.

Recently, more and more countries and R&D institutions announced their program on vaccine development against SARS-CoV-2. However, vaccine development has its own rules, and a successful SARS-CoV-2 vaccine could not be achieved overnight. After vaccine design and preparation, it will undergo efficacy and safety evaluation, quality standard establishment before entering clinical trials. Generally, three phases of clinical trials will be carried out to evaluate the safety, immunogenicity, and efficacy of the vaccine. Typically, the development of novel vaccines generally takes 10–20 years, and the success rate is less than 10%, even for a vaccine that enters clinical trials. In the past 30 years, the US FDA has approved a total of nearly 3,000 clinical trials on vaccine applications and less than 20 vaccines were approved for the market. Although the development, manufacture, and marketing of vaccines are supervised by strict regulations, and for the sake of human health, we must lay a solid foundation and conduct research in accordance with these scientific laws.

Recently, there is report that 149 sites of mutations were identified across the genome of 103 sequenced strains of SARS-CoV-2, and the virus had evolved into two subtypes, termed L and S subtype. The study also indicated that the two subtypes showed great differences in geographical distribution, transmission ability, and severity of disease, which add more difficulties for vaccine design [69].

Currently, clinical trials testing different drugs are ongoing and will possibly allow the identification of a potential drug to treat the disease associated to SARS-CoV-2, and the rapid development and application of a vaccine is a powerful means to prevent the global epidemic of COVID-19. Although vaccine development is slower than the spreading of the epidemic, it is still necessary and urgent. Firstly, the epidemic is still spreading all over the world and more and more confirmed cases and identified, and the inflection point has not yet been reached yet. Secondly, SARS-CoV-2 infection may become a flu-like seasonal disease and coexist with human beings for a long time [70].

It should be pointed out that SARS-CoV-2 was identified in no more than 3 months, related research on pathogenic characteristics and mechanisms of this novel coronavirus have just begun, and thus the data and information collected are also very limited and need to be continuously accumulated and documented. This review also provides the authors’ own opinions, and we hope it could provide useful information for colleagues.

## References

[1] Hui D.S., Azhar E., Madani T.A., Ntoumi F., Kock R., Dar O., Ippolito G., McHugh T.D., Memish Z.A., Drosten C. (2020). The continuing 2019-nCoV epidemic threat of novel coronaviruses to global health—The latest 2019 novel coronavirus outbreak in Wuhan, China. Int. J. Infect. Dis. IJID Off. Publ. Int. Soc. Infect. Dis..

[2] Zhu N., Zhang D., Wang W., Li X., Yang B., Song J., Zhao X., Huang B., Shi W., Lu R. (2020). A Novel Coronavirus from Patients with Pneumonia in China, 2019. N. Engl. J. Med..

[3] Guo Y.R., Cao Q.D., Hong Z.S., Tan Y.Y., Chen S.D., Jin H.J., Tan K.S., Wang D.Y., Yan Y. (2020). The origin, transmission and clinical therapies on coronavirus disease 2019 (COVID-19) outbreak—An update on the status. Mil. Med. Res..

[4] Phan T. (2020). Novel coronavirus: From discovery to clinical diagnostics. Infect. Genet. Evolut. J. Mol. Epidemiol. Evolut. Genet. Infect. Dis..

[5] Hoffmann M., Kleine-Weber H., Schroeder S., Kruger N., Herrler T., Erichsen S., Schiergens T.S., Herrler G., Wu N.H., Nitsche A. (2020). SARS-CoV-2 Cell Entry Depends on ACE2 and TMPRSS2 and Is Blocked by a Clinically Proven Protease Inhibitor. Cell.

[6] Huang C., Wang Y., Li X., Ren L., Zhao J., Hu Y., Zhang L., Fan G., Xu J., Gu X. (2020). Clinical features of patients infected with 2019 novel coronavirus in Wuhan, China. Lancet.

[7] Remy V., Largeron N., Quilici S., Carroll S. (2014). The Economic Value of Vaccination: Why Prevention Is Wealth. Value Health J. Int. Soc. Pharmacoecon. Outcomes Res..

[8] Barteling S.J. (2002). Development and performance of inactivated vaccines against foot and mouth disease. Revue Sci. Tech..

[9] Minor P.D. (2015). Live attenuated vaccines: Historical successes and current challenges. Virology.

[10] Marohn M.E., Barry E.M. (2013). Live attenuated tularemia vaccines: Recent developments and future goals. Vaccine.

[11] Wrapp D., Wang N., Corbett K.S., Goldsmith J.A., Hsieh C.L., Abiona O., Graham B.S., McLellan J.S. (2020). Cryo-EM structure of the 2019-nCoV spike in the prefusion conformation. Science.

[12] Lan J., Ge J., Yu J., Shan S., Zhou H., Fan S., Zhang Q., Shi X., Wang Q., Zhang L. (2020). Crystal structure of the 2019-nCoV spike receptor-binding domain bound with the ACE2 receptor. BioRxiv.

[13] Zhou Y., Jiang S., Du L. (2018). Prospects for a MERS-CoV spike vaccine. Expert Rev. Vaccines.

[14] Du L., He Y., Zhou Y., Liu S., Zheng B.J., Jiang S. (2009). The spike protein of SARS-CoV--a target for vaccine and therapeutic development. Nat. Rev. Microbiol..

[15] Zakhartchouk A.N., Sharon C., Satkunarajah M., Auperin T., Viswanathan S., Mutwiri G., Petric M., See R.H., Brunham R.C., Finlay B.B. (2007). Immunogenicity of a receptor-binding domain of SARS coronavirus spike protein in mice: Implications for a subunit vaccine. Vaccine.

[16] Woo P.C., Lau S.K., Tsoi H.W., Chen Z.W., Wong B.H., Zhang L., Chan J.K., Wong L.P., He W., Ma C. (2005). SARS coronavirus spike polypeptide DNA vaccine priming with recombinant spike polypeptide from Escherichia coli as booster induces high titer of neutralizing antibody against SARS coronavirus. Vaccine.

[17] He Y., Zhou Y., Liu S., Kou Z., Li W., Farzan M., Jiang S. (2004). Receptor-binding domain of SARS-CoV spike protein induces highly potent neutralizing antibodies: Implication for developing subunit vaccine. Biochem. Biophys. Res. Commun..

[18] Li F. (2016). Structure, Function, and Evolution of Coronavirus Spike Proteins. Ann. Rev. Virol..

[19] Pallesen J., Wang N., Corbett K.S., Wrapp D., Kirchdoerfer R.N., Turner H.L., Cottrell C.A., Becker M.M., Wang L., Shi W. (2017). Immunogenicity and structures of a rationally designed prefusion MERS-CoV spike antigen. Proc. Natl. Acad. Sci. USA.

[20] Coleman C.M., Liu Y.V., Mu H., Taylor J.K., Massare M., Flyer D.C., Smith G.E., Frieman M.B. (2014). Purified coronavirus spike protein nanoparticles induce coronavirus neutralizing antibodies in mice. Vaccine.

[21] Muthumani K., Falzarano D., Reuschel E.L., Tingey C., Flingai S., Villarreal D.O., Wise M., Patel A., Izmirly A., Aljuaid A. (2015). A synthetic consensus anti-spike protein DNA vaccine induces protective immunity against Middle East respiratory syndrome coronavirus in nonhuman primates. Sci. Transl. Med..

[22] Zhu X., Liu Q., Du L., Lu L., Jiang S. (2013). Receptor-binding domain as a target for developing SARS vaccines. J. Thorac. Dis..

[23] Lan J., Yao Y., Deng Y., Chen H., Lu G., Wang W., Bao L., Deng W., Wei Q., Gao G.F. (2015). Recombinant Receptor Binding Domain Protein Induces Partial Protective Immunity in Rhesus Macaques Against Middle East Respiratory Syndrome Coronavirus Challenge. EBioMedicine.

[24] Nyon M.P., Du L., Tseng C.K., Seid C.A., Pollet J., Naceanceno K.S., Agrawal A., Algaissi A., Peng B.H., Tai W. (2018). Engineering a stable CHO cell line for the expression of a MERS-coronavirus vaccine antigen. Vaccine.

[25] Jiang S., He Y., Liu S. (2005). SARS vaccine development. Emerg. Infect. Dis..

[26] Krempl C., Schultze B., Laude H., Herrler G. (1997). Point mutations in the S protein connect the sialic acid binding activity with the enteropathogenicity of transmissible gastroenteritis coronavirus. J. Virol..

[27] Promkuntod N., van Eijndhoven R.E., de Vrieze G., Grone A., Verheije M.H. (2014). Mapping of the receptor-binding domain and amino acids critical for attachment in the spike protein of avian coronavirus infectious bronchitis virus. Virology.

[28] Jiaming L., Yanfeng Y., Yao D., Yawei H., Linlin B., Baoying H., Jinghua Y., Gao G.F., Chuan Q., Wenjie T. (2017). The recombinant N-terminal domain of spike proteins is a potential vaccine against Middle East respiratory syndrome coronavirus (MERS-CoV) infection. Vaccine.

[29] Chen Y., Lu S., Jia H., Deng Y., Zhou J., Huang B., Yu Y., Lan J., Wang W., Lou Y. (2017). A novel neutralizing monoclonal antibody targeting the N-terminal domain of the MERS-CoV spike protein. Emerg. Microbes Infect..

[30] Wang Y., Tai W., Yang J., Zhao G., Sun S., Tseng C.K., Jiang S., Zhou Y., Du L., Gao J. (2017). Receptor-binding domain of MERS-CoV with optimal immunogen dosage and immunization interval protects human transgenic mice from MERS-CoV infection. Hum. Vaccines Immunother..

[31] Adney D.R., Wang L., van Doremalen N., Shi W., Zhang Y., Kong W.P., Miller M.R., Bushmaker T., Scott D., de Wit E. (2019). Efficacy of an Adjuvanted Middle East Respiratory Syndrome Coronavirus Spike Protein Vaccine in Dromedary Camels and Alpacas. Viruses.

[32] Alsaadi E.A.J., Neuman B.W., Jones I.M. (2019). A Fusion Peptide in the Spike Protein of MERS Coronavirus. Viruses.

[33] McBride R., van Zyl M., Fielding B.C. (2014). The coronavirus nucleocapsid is a multifunctional protein. Viruses.

[34] Leung D.T., Tam F.C., Ma C.H., Chan P.K., Cheung J.L., Niu H., Tam J.S., Lim P.L. (2004). Antibody response of patients with severe acute respiratory syndrome (SARS) targets the viral nucleocapsid. J. Infect. Dis..

[35] Kim T.W., Lee J.H., Hung C.F., Peng S., Roden R., Wang M.C., Viscidi R., Tsai Y.C., He L., Chen P.J. (2004). Generation and characterization of DNA vaccines targeting the nucleocapsid protein of severe acute respiratory syndrome coronavirus. J. Virol..

[36] Collisson E.W., Pei J., Dzielawa J., Seo S.H. (2000). Cytotoxic T lymphocytes are critical in the control of infectious bronchitis virus in poultry. Dev. Comp. Immunol..

[37] Seo S.H., Pei J., Briles W.E., Dzielawa J., Collisson E.W. (2000). Adoptive transfer of infectious bronchitis virus primed alphabeta T cells bearing CD8 antigen protects chicks from acute infection. Virology.

[38] Buchholz U.J., Bukreyev A., Yang L., Lamirande E.W., Murphy B.R., Subbarao K., Collins P.L. (2004). Contributions of the structural proteins of severe acute respiratory syndrome coronavirus to protective immunity. Proc. Natl. Acad. Sci. USA.

[39] Neuman B.W., Kiss G., Kunding A.H., Bhella D., Baksh M.F., Connelly S., Droese B., Klaus J.P., Makino S., Sawicki S.G. (2011). A structural analysis of M protein in coronavirus assembly and morphology. J. Struct. Biol..

[40] Pang H., Liu Y., Han X., Xu Y., Jiang F., Wu D., Kong X., Bartlam M., Rao Z. (2004). Protective humoral responses to severe acute respiratory syndrome-associated coronavirus: Implications for the design of an effective protein-based vaccine. J. Gener. Virol..

[41] Liu J., Sun Y., Qi J., Chu F., Wu H., Gao F., Li T., Yan J., Gao G.F. (2010). The membrane protein of severe acute respiratory syndrome coronavirus acts as a dominant immunogen revealed by a clustering region of novel functionally and structurally defined cytotoxic T-lymphocyte epitopes. J. Infect. Dis..

[42] Nieto-Torres J.L., DeDiego M.L., Verdia-Baguena C., Jimenez-Guardeno J.M., Regla-Nava J.A., Fernandez-Delgado R., Castano-Rodriguez C., Alcaraz A., Torres J., Aguilella V.M. (2014). Severe acute respiratory syndrome coronavirus envelope protein ion channel activity promotes virus fitness and pathogenesis. PLoS Pathog..

[43] Sharma A., Krause A., Worgall S. (2011). Recent developments for Pseudomonas vaccines. Hum. Vaccines.

[44] Takashima Y., Osaki M., Ishimaru Y., Yamaguchi H., Harada A. (2011). Artificial molecular clamp: A novel device for synthetic polymerases. Angew. Chem..

[45] Liu H., Su D., Zhang J., Ge S., Li Y., Wang F., Gravel M., Roulston A., Song Q., Xu W. (2017). Improvement of Pharmacokinetic Profile of TRAIL *via* Trimer-Tag Enhances its Antitumor Activity in vivo. Sci. Rep..

[46] Pardi N., Hogan M.J., Porter F.W., Weissman D. (2018). mRNA vaccines—A new era in vaccinology. Nat. Rev. Drug Discov..

[47] Jahanafrooz Z., Baradaran B., Mosafer J., Hashemzaei M., Rezaei T., Mokhtarzadeh A., Hamblin M.R. (2020). Comparison of DNA and mRNA vaccines against cancer. Drug Discov. Today.

[48] Liu M.A. (2019). A Comparison of Plasmid DNA and mRNA as Vaccine Technologies. Vaccines.

[49] Gonzalez-Nicolini V., Sanchez-Bustamante C.D., Hartenbach S., Fussenegger M. (2006). Adenoviral vector platform for transduction of constitutive and regulated tricistronic or triple-transcript transgene expression in mammalian cells and microtissues. J. Gene Med..

[50] Azmi F., Ahmad Fuaad A.A., Skwarczynski M., Toth I. (2014). Recent progress in adjuvant discovery for peptide-based subunit vaccines. Hum. Vaccines Immunother..

[51] Ahmed S.F., Quadeer A.A., McKay M.R. (2020). Preliminary identification of potential vaccine targets for 2019-nCoV based on SARS-CoV immunological studies. BioRxiv.

[52] Bao L., Deng W., Huang B., Gao H., Ren L., Wei Q., Yu P., Xu Y., Liu J., Qi F. (2020). The Pathogenicity of 2019 Novel Coronavirus in hACE2 Transgenic Mice. BioRxiv.

[53] Noranate N., Takeda N., Chetanachan P., Sittisaman P., A-nuegoonpipat A., Anantapreecha S. (2014). Characterization of chikungunya virus-like particles. PLoS ONE.

[54] Dong H., Guo H.C., Sun S.Q. (2014). Virus-like particles in picornavirus vaccine development. Appl. Microbiol. Biotechnol..

[55] Temperton N.J., Chan P.K., Simmons G., Zambon M.C., Tedder R.S., Takeuchi Y., Weiss R.A. (2005). Longitudinally profiling neutralizing antibody response to SARS coronavirus with pseudotypes. Emerg. Infect. Dis..

[56] Temperton N.J., Hoschler K., Major D., Nicolson C., Manvell R., Hien V.M., Ha do Q., de Jong M., Zambon M., Takeuchi Y. (2007). A sensitive retroviral pseudotype assay for influenza H5N1-neutralizing antibodies. Influenza Respir. Viruses.

[57] Hogenesch H. (2012). Mechanism of immunopotentiation and safety of aluminum adjuvants. Front. Immunol..

[58] Tsai T.F. (2013). Fluad(R)-MF59(R)-Adjuvanted Influenza Vaccine in Older Adults. Infect. Chemother..

[59] Kensil C.R. (1996). Saponins as vaccine adjuvants. Crit. Rev. Ther. Drug Carr. Syst..

[60] Didierlaurent A.M., Laupeze B., Di Pasquale A., Hergli N., Collignon C., Garcon N. (2017). Adjuvant system AS01: Helping to overcome the challenges of modern vaccines. Expert Rev. Vaccines.

[61] Garcon N., Van Mechelen M. (2011). Recent clinical experience with vaccines using MPL- and QS-21-containing adjuvant systems. Expert Rev. Vaccines.

[62] Garcon N., Vaughn D.W., Didierlaurent A.M. (2012). Development and evaluation of AS03, an Adjuvant System containing alpha-tocopherol and squalene in an oil-in-water emulsion. Expert Rev. Vaccines.

[63] Petrovsky N. (2015). Comparative Safety of Vaccine Adjuvants: A Summary of Current Evidence and Future Needs. Drug Saf..

[64] Jiang S. (2020). Don’t rush to deploy COVID-19 vaccines and drugs without sufficient safety guarantees. Nature.

[65] Luo F., Liao F.L., Wang H., Tang H.B., Yang Z.Q., Hou W. (2018). Evaluation of Antibody-Dependent Enhancement of SARS-CoV Infection in Rhesus Macaques Immunized with an Inactivated SARS-CoV Vaccine. Virolog. Sin..

[66] Wang Q., Zhang L., Kuwahara K., Li L., Liu Z., Li T., Zhu H., Liu J., Xu Y., Xie J. (2016). Immunodominant SARS Coronavirus Epitopes in Humans Elicited both Enhancing and Neutralizing Effects on Infection in Non-human Primates. ACS Infect. Dis..

[67] Liu L., Wei Q., Lin Q., Fang J., Wang H., Kwok H., Tang H., Nishiura K., Peng J., Tan Z. (2019). Anti-spike IgG causes severe acute lung injury by skewing macrophage responses during acute SARS-CoV infection. JCI Insight.

[68] Ontiveros E., Kim T.S., Gallagher T.M., Perlman S. (2003). Enhanced virulence mediated by the murine coronavirus, mouse hepatitis virus strain JHM, is associated with a glycine at residue 310 of the spike glycoprotein. J. Virol..

[69] Tang X., Wu C., Li X., Song Y., Yao X., Wu X., Duan Y., Zhang H., Wang Y., Qian Z. (2020). On the origin and continuing evolution of SARS-CoV-2. Natl. Sci. Rev..

[70] Neher R.A., Dyrdak R., Druelle V., Hodcroft E.B., Albert J. (2020). Potential impact of seasonal forcing on a SARS-CoV-2 pandemic. MedRxiv.

